# Research on Design Parameters for Fatigue Performance of Asphalt Mixtures

**DOI:** 10.3390/ma17205048

**Published:** 2024-10-16

**Authors:** Yunlong Shang, Hongyu Han, Wenwen Feng, Xinyu Cong, Yiqiu Tan

**Affiliations:** 1 School of Transportation Science and Engineering, Harbin Institute of Technology, Harbin 150090, China; shangyunlong2024@163.com (Y.S.); 22s032025@stu.hit.edu.cn (H.H.); fengwenwen2024@126.com (W.F.); congxinyu@hit.edu.cn (X.C.); 2 Engineering Research Center of Airport, Civil Aviation Administration of China, 111 North Fourth Ring East Road, Chaoyang District, Beijing 100029, China; 3 State Key Laboratory of Urban Water Resource and Environment, Harbin Institute of Technology, Harbin 150090, China

**Keywords:** asphalt mixture, fatigue performance, strain rate, design method

## Abstract

The fatigue performance of the asphalt mixture was the main focus of this study, with five typical factors—phase angle, cumulative dissipated energy, failure strain, failure stiffness modulus, and strain rate—identified as potential design indexes. The effect of asphalt content on the parameters under different gradation and stress ratios was tested. It was observed that the selected parameters exhibited varying levels of sensitivity and relevance to the fatigue behavior of asphalt mixtures under cyclic loads. By comparison, the strain rate proved sensitive to the asphalt content and independent of the other parameters, namely aggregate gradations and stress ratio, thus establishing the strain rate as a critical design index based on fatigue performance. On this basis, a design method based on the fatigue performance for the asphalt mixtures is herein proposed. It was confirmed that the asphalt mixture formulated using the proposed method exhibited enhanced fatigue endurance compared to those designed using the conventional method.

## 1. Introduction

Fatigue performance constitutes a critical aspect of asphalt mixtures, with potential pavement failures often stemming from inadequate fatigue endurance, such as fatigue cracking, rutting, and thermal cracking. The method used to analyze the fatigue performance of asphalt mixtures relies predominantly on phenomenological experience, mechanical analysis, and energy deduction. The phenomenological approach is grounded in empirical results [1]; the fracture mechanics approach specifically targets crack initiation and propagation [2]; and the dissipated energy approach examines both deformation and failure [3].

The first model of strain evolution based on cyclic loads was proposed in the 1980s, which was a numerical relationship derived from experience without physical explanation [4]. This model describes the relationship between the reciprocal of strain and the cycle of loads, with parameters empirically established through testing. Although the model has been refined by incorporating additional parameters, the underlying mechanisms of failure due to fatigue remain undisclosed. A variety of experimental techniques are employed to study the fatigue behavior of asphalt mixtures, including four-point bending beam [5], indirect tensile (splitting tensile) [6], cyclic uniaxial tension–compression [7], semi-circular bending (SCB) [8], and a range of other monotonic and cyclic fatigue tests [9].

In order to express the fatigue properties in physical terms, fracture mechanics and damage theory in a continuous matrix are used to illustrate fatigue damage in asphalt-based materials. Paris and Erdogan [10] proposed a model to elucidate crack propagation in the asphalt mixture. In this model, the ratio of crack length to the cycle of load is a constant influenced by the material properties, specimen dimensions, and boundary conditions. Kim et al. [11] posited that fatigue damage in asphalt-based materials was triggered by cyclic loads and the fatigue strain interfered with creep, which endowed the failure mechanism related to fatigue with scientificity and rationality. The theory of fracture mechanics and damage evolution is complex, making its widespread application challenging. The viscoelasticity of asphalt helps to consume energy before the fracture of the asphalt mixture [12]. Research into the relationship between fatigue life and energy has shown that the energy consumed during cyclic loading serves as a parameter for assessing the fatigue life of asphalt-based materials and exhibits an exponential relationship with the energy dissipated [13]. Although the energy consumed under cyclic loads acts as a theoretical index and appears positively related to fatigue life, it remains a dependent index, influenced by the material’s properties and test parameters [14]. Consequently, using dissipated energy as a parameter for assessing fatigue life has its limitations.

It is expected that a well-designed asphalt mixture will withstand major distresses in flexible pavements. Asphalt mixtures used in the road base and stress-absorbing layers require excellent fatigue performance to prevent reflection cracks. Actually, an asphalt mixture with long fatigue endurance needs to be designed based on the specific performance. The Marshall method, recommended as the current design method by JTG F40-2004 [15], focuses on the mechanical and physical properties of asphalt mixtures to ensure competent pavement performance. However, the fatigue performance of asphalt mixtures is not considered during the design phase and can only be assessed through testing after the material has been designed. It is meaningful to find a suitable index parallel to the Marshall stability (MS), which is used to build an asphalt mixture design method based on fatigue performance.

In this study, parameters related to fatigue performance were identified from commonly used fatigue performance evaluation indexes for asphalt mixtures. The relationship between these parameters and the asphalt content was analyzed, and the most sensitive parameters were chosen as design indicators. On this basis, a design method based on fatigue performance was proposed, and its effectiveness was verified by comparing it with the conventional design method.

## 2. Materials and Experiments

### 2.1. Components

Two types of commercially available asphalt—SBS modified asphalt (SBS-AP) and high viscosity and elasticity modified asphalt (VE-AP)—sourced from China National Offshore Oil Corporation were utilized in this study. The basic technical properties of the asphalt are presented in Table 1. Limestone powder was used as the filler in the asphalt mixture. The fine and coarse aggregates used were Andesite that had been sieved according to the gradation.

### 2.2. Mix Design

**Figure 1:**
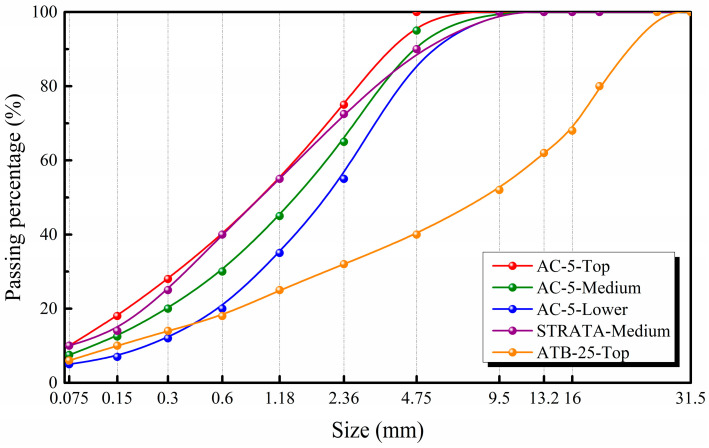
Size Distribution of the Five Aggregate Grading Selected.

The materials used to resist fatigue in asphalt pavements are usually fine-grained asphalt mixtures. Three types of asphalt mixtures—AC-5, STRATA, and ATB-25—were prepared for testing. The gradation ranges for the three asphalt mixtures were determined in accordance with JTG F40-2004 [15]. In particular, three gradations for AC-5 were determined according to the upper limit, median, and lower limit of the gradation range. For STRATA and ATB-25, gradations were assigned based on the median and upper limit of the respective gradation ranges. The gradation curves for the asphalt mixtures are depicted in Figure 1.

For each gradation, four to five different asphalt content levels were selected, with a gradient range of 0.3–0.5%. Asphalt content ranged from 8.0 to 9.5% for AC-5, 7.5 to 9.0% for STRATA, and 3.5 to 4.6% for ATB-25. Two types of modified asphalt were used as described above: VE-AP for the AC-5-Medium gradation, and SBS-AP for the other gradations (AC-5-T, AC-5-L, STRATA, and ATB-25). Table 2 presents the volumetric parameters of asphalt mixtures formulated using the selected gradations and varying asphalt contents. All asphalt mixtures in this study were molded using a gyratory compactor manufactured by Pine Instrument Co, Grove City, PA, USA.

### 2.3. Experimental Procedures

Splitting tests were conducted to obtain the splitting strength, which was used to determine the stress level for the fatigue tests. The splitting tests were carried out in accordance with JTG E20-2011 [16], and the vertical deformation of the specimen was documented. The dimensions of the test specimen for the splitting test conformed to those used for the Marshall test, specifically a cylinder with a diameter of 101.6 mm and a height of 63.5 mm. Two steel modules with a width of 12.7 mm and a curvature radius of 50.8 mm were secured on each end of a specimen to focus the vertical load.

The fatigue behavior of the asphalt mixture under tensile load was characterized using the splitting test, as this test can reflect the stress state of a practical pavement structure to some extent. Stress level was utilized as a control parameter for the fatigue load in this study. Loads were applied using a successive half-sine wave model, and a loading frequency of 10 Hz was set for the test, equivalent to a driving speed of 60–65 km/h on the road. The peak stress of the half-sine wave was determined according to Equations (1) and (2):

(1) $$\sigma_{p}=n\cdot \sigma_{t,s}$$

(2) $$\sigma_{t,s}=\frac{2P_{\max}}{\pi \cdot d\cdot l}$$

where $\sigma_{p}$ (MPa) and $\sigma_{t,s}$ (MPa) represent the peak stress of the half-sine wave and the splitting tensile strength of the specimen, respectively; n represents the stress level for the test. In this study, the stress level for the fatigue performance test was assigned as 0.3, 0.4, and 0.6; $P_{\max}$ (N) is the maximum load for the splitting tensile test, and d (mm) and l (mm) are the diameter and height of the cylinder specimen.

The test temperature was maintained at 15 ± 0.5 °C, and the specimens were conditioned in a water bath for 4 h prior to testing to achieve the required temperature. The Digital Image Correlation method (DIC) was employed to measure specimen deformation during the test, playing a crucial role in the subsequent analysis of fatigue strain. The tensile strain and stiffness modulus of the specimen at the failure moment were derived from Equations (3)–(5) according to the JTG E20-2011 [16]:

(3) $$\varepsilon_{f}=X_{f}\cdot (0.0307+0.0936\mu )/(1.35+5\mu )$$

(4) $$S_{f}=P_{\max}\cdot (0.27+1.0\mu )/(d\cdot X_{f})$$

(5) $$\mu =(0.135a-1.794)/(-0.5a-0.0314),\;a=Y_{f}/X_{f}$$

where $\varepsilon_{f}$ and $S_{f}$ (MPa) are the tensile strain and stiffness modulus of the specimen at the failure moment, respectively; $X_{f}$ (mm) and $Y_{f}$ (mm) are the horizontal and vertical deformation of the specimen at the failure moment, respectively; and μ is the Poisson’s ratio. In order to evaluate the fatigue performance of the asphalt mixture, the phase angle, dissipated energy within one circle of the half-sine wave load, and total dissipated energy within the whole loading period were analyzed according to Equations (6)–(8):

(6) ϕ=360f⋅t

(7) $$E_{i}=\pi \cdot \sigma_{n}\cdot \varepsilon_{n}\cdot \sin\phi$$

(8) $$E_{f}=\sum E_{i}$$

where ϕ (rad), f (Hz), and t (s) represent the phase angle, loading frequency, and hysteretic time between the stress peak and the strain peak; $E_{i}$ (MJ/m^3^) and $E_{f}$ (MJ/m^3^) represent the dissipated energy within one circle of the half-sine wave load and the total dissipated energy; and $\sigma_{n}$ (MPa) and $\varepsilon_{n}$ represent the tensile stress and strain at a certain stress level.

**Figure 2:**
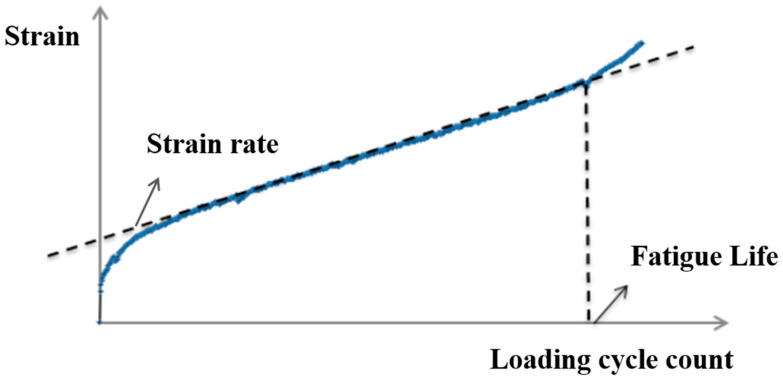
Relationship between Strain and Loading Cycle Count of Asphalt Mixtures in Stress Control Mode.

The failure of asphalt mixtures under cyclic loads occurred when the tensile strain evolved from a linear increase into a nonlinear trend with time [17]. The relationship between strain and loading cycle count is depicted in Figure 2. In this study, the slope of the linear segment is defined as the strain rate ($\dot{\varepsilon }$), which reflected the level of strain increment during the fatigue performance test.

For each specimen type in this study, three identical specimens were prepared. Average test results were employed for qualitative analysis, while all test results were utilized to conduct correlation analysis using the Pearson correlation coefficient (PCC). The PCC was applied to ascertain the relevance of parameters to fatigue performance. The PCC is derived from Equation (9):

(9) $$\rho_{\mathrm{XY}}=\frac{Cov(X,Y)}{\sqrt{D(X)}\sqrt{D(Y)}}=\frac{E((X-EX)(Y-EY))}{\sqrt{D(X)}\sqrt{D(Y)}}$$

where $\rho_{XY}$ is the PCC; Cov(X,Y) is the covariance of X and Y; D(X) and E(X) are square deviation and expectations of X, respectively; and the meaning is the same for D(Y) and E(Y). The absolute value of PCC close to 1.0 represents the strong relevance between the two corresponding factors.

## 3. Results

### 3.1. Effect of Stress Level on the Fatigue Behavior

Specimens fabricated with AC-5 median gradation and varying asphalt contents were tested across three stress levels; the results are presented in Table 3. Initially, the phase angle decreased but subsequently increased as asphalt content rose, recording the lowest value at an 8.5% asphalt content across all stress levels. The phase angle also rose with increasing stress levels; for example, specimens at a 0.6 stress level exhibited a higher phase angle (approximately 0.25 rad) compared to those at 0.3 and 0.4 at lower stress levels. As stress increased, variations in phase angle attributable to changes in asphalt content became less pronounced. Notably, specimens subjected to the highest stress levels failed more rapidly than those at lower levels.

Similarly, dissipated energy fluctuated with asphalt content, peaking at the lowest stress level (0.3). With increasing asphalt content, a pattern of initial decrease followed by an increase and subsequent decrease was observed. This pattern reached a peak at an asphalt content of 9.0%, where the dissipated energy was 1.963 MJ/m^3^, and recorded its lowest value at 8.5%, with a dissipated energy of 1.273 MJ/m^3^. Above a stress level of 0.3, dissipated energy values stabilized and increased with higher asphalt content. However, with increasing stress ratios, variations in cumulative dissipated energy became less pronounced. According to Equation (7), specimens subjected to higher stress levels dissipated more energy per half-sine wave load, attributed to the increased peak stress, strain, and phase angle. Despite increased energy consumption per cycle, the total dissipated energy deviated from theoretical expectations. This occurred because higher stress levels led to greater energy consumption per loading cycle, yet the total energy dissipated remained capped, resulting in fewer loading cycles for the specimen and thus a shorter fatigue life.

The tensile strain at the failure moment increased monotonically with asphalt content, thereby enhancing deformability, with ultimate strains ranging from 0.012 at lower contents to 0.022 at higher levels. Nevertheless, across different stress levels, the ultimate tensile strains for specimens with identical asphalt contents were notably similar. Thus, deformability appeared to be influenced more by the specific mix design, including aggregate gradation and asphalt content, than by the magnitude of the applied load. Conversely, the stiffness modulus demonstrated a decreasing trend with increasing asphalt content, indicating reduced structural rigidity and heightened fatigue damage at higher asphalt percentages. For instance, at an asphalt content of 9.5%, the stiffness modulus varied significantly across stress levels, having increased from 34.96 MPa at a stress ratio of 0.3 to 71.69 MPa at 0.6.

The strain rate demonstrated a pattern, initially decreasing and then increasing with higher asphalt content at constant stress levels, and was notably influenced by the applied load. For example, at the highest stress level of 0.6, the average strain rate reached 65 × 10^−6^/s, being up to 15 and 3 times greater than at stress levels of 0.3 and 0.4, respectively. The strain rate, a dynamic parameter reflecting pre-failure strain increments, diminished as asphalt content increased up to a certain threshold, owing to rising cohesive and embedded forces within the mixture. Beyond this point, the mixture softened, resulting in increased fatigue rate and strain rate. Additionally, short dynamic loading cycles, incapable of accommodating recovery from hysteresis effects, led to rapid residual strain accumulation as stress levels rose. This led to an increase in the strain rate with the stress ratio.

**Figure 3:**
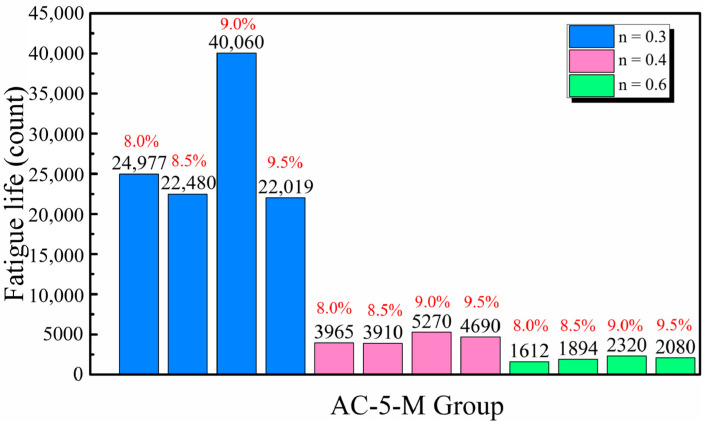
Effect of Asphalt Content and Stress Levels on the Fatigue Life of AC-5-M Asphalt Mixtures.

In a stress-controlled mode fatigue-splitting test, both displacement and the number of cyclic load applications can be obtained during the test. Fatigue life is defined as the total number of load cycles an asphalt mixture specimen withstands until fatigue failure occurs. The fatigue life for AC type specimens is depicted in Figure 3. Fatigue life varied with stress level and asphalt content; increased asphalt enhanced cohesive performance up to a threshold, beyond which excess asphalt led to early deformation under load. The stiffness modulus, which indicates deformation recovery capacity under specific loads and temperatures, decreased with higher ultimate strains but increased with stress levels, thus impacting fatigue life. Additionally, higher stress levels accelerated strain accumulation and increased energy consumption per cycle but reduced overall fatigue life due to fewer cycles and limited total energy consumption, thereby making materials more susceptible to fatigue damage.

### 3.2. Effect of Aggregate Gradations on the Fatigue Behavior

Specimens with various aggregate gradations and asphalt contents were tested at a stress level of 0.3, with results for splitting tensile strength and fatigue performance detailed in Table 4 and Table 5. The phase angles varied slightly with changes in asphalt content. For AC-5-T and AC-5-L, phase angles peaked at asphalt contents of 9.0% (0.1336 rad) and 8.5% (0.1641 rad), respectively, and then decreased. In contrast, for STRATA and ATB-25, phase angles first decreased then increased, reaching their lowest values at asphalt contents of 8.5% (0.1277 rad) and 4.3% (0.1436 rad), respectively.

**Figure 4:**
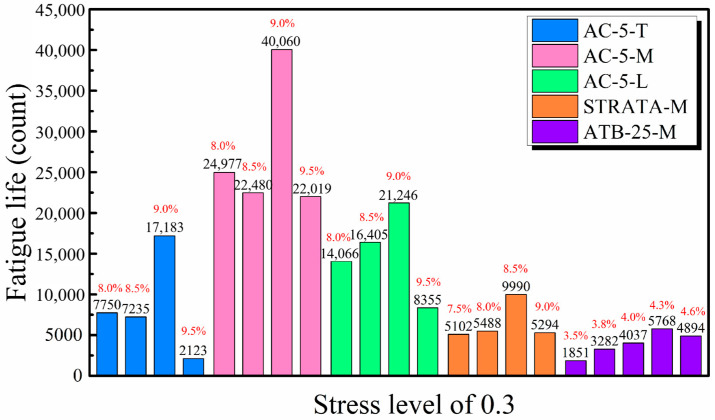
Effect of Aggregate Gradation and Asphalt Content on Fatigue Life at a Stress Level of 0.3.

Specimens with varied gradations exhibited significant differences in dissipated energy, which fluctuated according to asphalt content. This energy dissipation, influenced by particle redistribution and the structural makeup of the asphalt mix, directly impacted fatigue life, with longer fatigue life correlating with higher energy consumption during cyclic loads. As depicted in Figure 4, those with fine aggregates such as AC-5 and STRATA consumed less energy. For AC-5-T, energy dissipation exhibited a pattern of increasing, decreasing, and then increasing again, peaking at an asphalt content of 8.5% (0.7314 MJ/m^3^) and dipping at 9.0% (0.1667 MJ/m^3^). Conversely, AC-5-L and STRATA exhibited peaks at asphalt contents of 9.0% and 8.5%, respectively. ATB-25 specimens, containing coarser aggregates, required more energy, with energy consumption peaking at 4.3% (0.8371 MJ/m^3^) and decreasing at 4.0% asphalt content (0.5170 MJ/m^3^).

The strain at failure was significantly influenced by aggregate gradations, with ATB-25-M, containing coarse aggregates, demonstrating a substantially lower ultimate strain of only 0.011—up to half that of other specimens. This specimen also exhibited a higher stiffness modulus, ranging from 113.6 to 261.2 MPa, attributable to the stiff constraint formed by interlocked coarse aggregates, which effectively limited asphalt movement under load. Although coarse aggregates play a crucial role in reducing deformation from the applied load, the control over the accumulation of residual strain from cyclic loads was minimal. The specimen with ATB-25-M gradation exhibited the highest strain rate, 17.21 × 10^−6^/s, slightly higher than that of the specimen without coarse aggregate gradation. Consequently, ATB-25-M did not achieve prolonged fatigue life despite controlled strain levels during cyclic loads. Figure 4 indicates that the optimum asphalt content for AC-5 and STRATA gradations, optimized for fatigue performance, is 9.0% and 8.5%, respectively, whereas ATB-25-M demonstrated inferior fatigue performance.

Figure 4 illustrates that fatigue life varies with aggregate gradation and asphalt content, generally increasing before decreasing as asphalt content changes. AC-5-L gradation demonstrated the best fatigue performance, with a lifespan ranging from 8000 to 22,000 cycles, followed by AC-5-T, which ranged from 2000 to 17,000 cycles. STRATA and ATB-25 gradations exhibited lower performances, with lifespans ranging from 5000 to 10,000 cycles and 1000 to 5000 cycles, respectively.

Zhang [18] investigated the variation in damage paths of asphalt mixtures with different gradations. From a microscopic perspective, fatigue damage typically began at the junction between coarse aggregate and asphalt mastic, and the fatigue life of asphalt mixtures was closely correlated with the size and content of the graded coarse aggregate as well as the asphalt content. Among the gradations selected for this study, in ATB-25, a higher coarse aggregate content enhanced the stiffness modulus of the mix and reduced the strain at failure; however, premature cracking at the junction between the coarse aggregate and asphalt mastic resulted in a reduced fatigue life. In contrast, AC-5 featured a lower coarse aggregate content, uniform aggregate distribution, and more uniform stress distribution, thereby extending fatigue life by mitigating local stress concentrations. In terms of asphalt content, initially, increasing asphalt content extended fatigue life by enhancing the embedding and cohesive forces within the asphalt mixtures. However, when the asphalt content exceeded a critical threshold, the mixture softened, and its fatigue resistance became overly reliant on cohesive forces, resulting in accelerated strain accumulation and ultimately reducing fatigue life. Therefore, the gradation of the AC and dense classes represented a superior choice for enhancing fatigue performance.

## 4. Discussion

Table 5 illustrates the fatigue behavior of the asphalt mixture, emphasizing the impact of asphalt content and aggregate gradation on various design parameters, with a detailed sensitivity analysis presented in Table 6, Table 7, Table 8 and Table 9. While the phase angle’s sensitivity to asphalt content varied, the cumulative dissipated energy, destructive strain, and strength modulus demonstrated varying degrees of sensitivity based on gradation. In contrast, the strain rate was sensitive to changes in asphalt content, particularly under varying stress levels and gradations, indicating its suitability as a fatigue design index. In conclusion, it is reasonable to adopt the strain rate of change as the fatigue design index for asphalt mixtures within the scope of this study.

Table 10 and Table 11 display the test results for strain rate and fatigue life, incorporating standard deviation (SD) and coefficient of variation (CV) to confirm the reproducibility and consistency of the data. According to Equation (9), the PCC for the strain rate and fatigue life of the asphalt mixtures are detailed in Table 12. As indicated in Table 12, all of the PCCs were negative, demonstrating that the strain rate inversely correlates with fatigue life. Across various stress levels and gradation types, the strain rate could predict the fatigue performance of the asphalt mixture to some degree.

**Figure 5:**
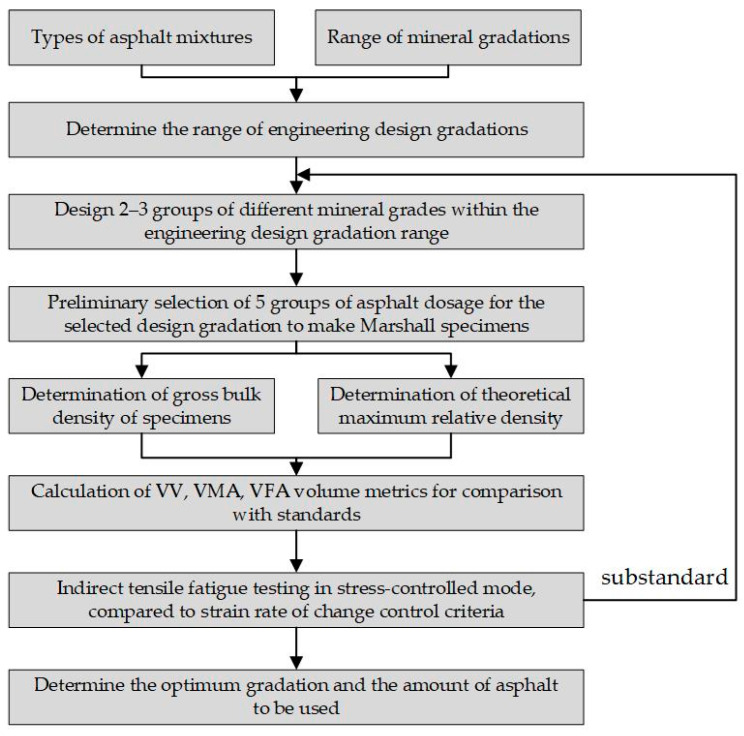
Flowchart of the Fatigue Performance-based Asphalt Mixture Design Process.

A fatigue performance-based asphalt mixture design method was introduced to meet fatigue endurance requirements, as depicted in Figure 5. This method aligns with standard asphalt mixture procedures but substitutes the Marshall test with a splitting test under cyclic loads to measure the strain rate.

To determine the optimal asphalt content and mix gradation, relationships between key properties—gross bulk density, void ratio, mineral gap ratio, asphalt saturation, and strain rate change—and asphalt content were analyzed. Points $a_{1}$, $a_{2}$, $a_{3}$, and $a_{4}$, corresponding to maximum density, target void ratio, minimum strain rate change, and mid-range asphalt saturation, respectively, were identified on these plots. The average of these values, calculated according to Equation (10), was defined as ${OAC}_{1}$. If this range did not encompass the required asphalt saturation, Equation (11) was applied instead. The median asphalt content between ${OAC}_{\min}$ and ${OAC}_{\max}$, satisfying all technical standards, was designated as ${OAC}_{2}$. The final optimum asphalt content (OAC) was established as the median of ${OAC}_{1}$ and ${OAC}_{2}$. If the mixture performance fell short of standards, adjustments to gradation and content were repeated until satisfactory results were achieved. A confirmatory test was conducted to validate the design method, with details of specific aggregate gradation provided in Table 13.

(10) $${OAC}_{1}=(a_{1}+a_{2}+a_{3}+a_{4})/4$$

(11) $${OAC}_{1}=(a_{1}+a_{2}+a_{3})/3$$

(12) $${OAC}_{2}=({OAC}_{\min}+{OAC}_{\max})/2$$

(13) $$OAC=({OAC}_{1}+{OAC}_{2})/2$$

where OAC represents the optimum asphalt content; ${OAC}_{1}$ represents the average of $a_{1}$, $a_{2}$, $a_{3}$, and $a_{4}$; ${OAC}_{2}$ represents the median of the range of asphalt content from ${OAC}_{\min}$ to ${OAC}_{\max}$ that meets all technical standards; $a_{1}$ represents the asphalt content corresponding to the maximum gross bulk density; $a_{2}$ represents the asphalt content corresponding to the target void ratio; $a_{3}$ represents the asphalt content corresponding to the minimum strain rate of change; $a_{4}$ represents the asphalt content corresponding to the mid-range value of asphalt saturation.

The target asphalt mixture was designed for the stress-absorbing layer in a pavement structure, which requires excellent fatigue performance. Marshall specimens, with asphalt contents ranging from 7.5% to 9.5% in 0.5% increments, were fabricated for conventional testing, while specimens with asphalt contents from 7.9% to 9.1% in 0.3% increments were prepared for the proposed method tests. The indexes for the different methods and their corresponding test results are displayed in Table 14 and Table 15. According to the JTG F40-2004 [15], the optimum asphalt content for the aggregate gradation was established at 8.4% using the conventional Marshall method. The optimum asphalt content for the same aggregate gradation changed when the design method was based on fatigue performance (strain rate method). As previously discussed, a low strain rate was beneficial in prolonging the fatigue life of the asphalt mixture. Thus, according to the results in Table 15, the optimum asphalt content ranged between 8.5% and 8.8%. Considering the remaining indexes, the optimum asphalt content for achieving excellent fatigue performance in the mixture was determined to be 8.7%.

**Figure 6:**
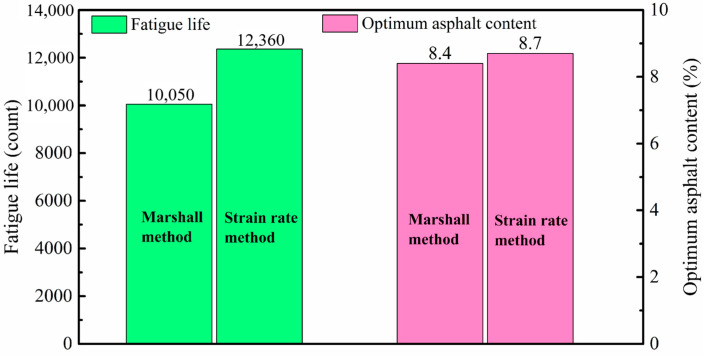
Fatigue Life Comparison of Asphalt Mixtures Designed Using the Marshall Method and the Strain Rate Method.

The fatigue life of the asphalt mixtures, designed using both the conventional and the proposed methods, is depicted in Figure 6. The asphalt mixture, designed via the strain rate method with an asphalt content of 8.7%—only 0.3% higher than its counterpart designed by the Marshall method—achieved a 12,360-count fatigue life, marking a 23% improvement over the mixture designed by the Marshall method. This comparison demonstrated that the strain rate is relevant to fatigue performance, and incorporating the strain rate as one of the indexes achieves a design that optimizes asphalt mixture fatigue performance.

## 5. Conclusions

This study investigated the fatigue performance of various asphalt mixtures under cyclic loading to clarify the relationship between their typical properties and fatigue performance. The primary focus was on examining how different stress levels and aggregate gradations influence the fatigue behavior of asphalt mixtures, aimed at selecting optimal design indexes. The main conclusions are as follows.

The fatigue behavior of asphalt mixtures under cyclic loading was found to be closely associated with stress levels, significantly affecting their properties. Elevated stress levels accelerated damage accumulation, consequently reducing the fatigue life of the mixtures. The parameters related to deformation, namely ultimate strain and strain rate, were relatively independent and effectively represented the fatigue performance without interference. Experimental results confirmed that finer aggregates significantly enhanced fatigue resistance by uniformly distributing stresses throughout the mix. Additionally, the test results showed that the optimal asphalt contents for AC-5 and STRATA gradations to achieve the best fatigue performance were 9.0% and 8.5%, respectively.

Sensitivity analysis identified the strain rate as the parameter most responsive to changes in asphalt content, with its variations closely correlating with alterations in fatigue life. This suggests that strain rate can serve as a predictive index for the fatigue performance of asphalt mixtures. Based on these insights, a novel asphalt mixture design method was developed, emphasizing fatigue performance and prioritizing strain rate over traditional Marshall stability for determining optimal asphalt content. Comparative experiments revealed that the optimal asphalt content determined by this new method was 8.7%, slightly higher than the 8.4% established by the conventional Marshall method. This adjustment led to a 23% enhancement in fatigue life, underscoring the efficacy of the new design approach in improving the fatigue performance of asphalt mixtures.

## Figures and Tables

**Table 1:** The Physical Properties of the Asphalt.

Test	SBS-AP	VE-AP
Penetration, 100 g, 5 s, 25 °C (0.1 mm)	67.2	89.0
Ductility, 5 cm/min, 5 °C (cm)	33.0	30.2
Softening point, T_R&B_ (°C)	63.7	86.0

Note: The penetration, ductility and softening point indexes listed in Table 1 were tested according to the JTG E20-2011 [16].

**Table 2:** Material Compositions of Tested Mineral–Asphalt Mixes.

Gradation	Asphalt Content (%)	Bulk Density (g/cm^3^)	VV (%)	VMA (%)	VFA (%)	Filler-to-Binder Ratio
AC-5-T	8.0	2.36	3.78	21.29	82.25	1.15
	8.5	2.38	2.31	21.11	89.06	1.08
	9.0	2.39	1.37	21.34	93.58	1.01
	9.5	2.37	1.16	22.18	94.78	0.95
AC-5-M	8.0	2.40	2.12	19.93	89.37	0.86
	8.5	2.39	1.81	20.69	91.37	0.81
	9.0	2.39	1.32	21.30	93.80	0.76
	9.5	2.38	1.06	22.10	95.22	0.71
AC-5-L	8.0	2.36	3.76	21.28	82.35	0.58
	8.5	2.37	2.84	21.53	86.84	0.54
	9.0	2.38	1.54	21.47	92.85	0.51
	9.5	2.37	1.23	22.24	94.52	0.48
STRATA-M	7.5	2.39	3.40	20.35	83.29	1.23
	8.0	2.40	2.30	20.46	88.77	1.15
	8.5	2.40	1.56	20.87	92.54	1.08
	9.0	2.39	1.30	21.65	93.98	1.01
ATB-25-T	3.5	2.49	4.71	11.24	58.35	1.65
	3.8	2.50	4.35	11.19	61.16	1.52
	4.0	2.52	2.93	10.74	72.80	1.44
	4.3	2.52	2.33	10.89	78.80	1.34
	4.6	2.54	1.27	10.62	88.04	1.24

Note: The label for gradation consists of two parts: the gradation type (the front part) and the gradation range information (the rear part); the capital letters T, M, and L in the label represent the top limit, medium value, and lower limit of the corresponding gradation range.

**Table 3:** Fatigue Test Results for AC-5-M Asphalt Mixtures at Varying Asphalt Contents and Stress Levels.

Label	n	Asphalt Content (%)	ϕ (rad)	$E_{f}$ (MJ/m^3^)	$\varepsilon_{f}$	$S_{f}$ (MPa)	$\dot{\varepsilon }\times 10^{-6}$ (s^−1^)
AC-5-M-8.0-3	0.3	8.0	0.160	1.455	0.012	64.276	3.323
AC-5-M-8.5-3		8.5	0.150	1.273	0.015	51.538	4.300
AC-5-M-9.0-3		9.0	0.153	1.963	0.015	37.574	3.038
AC-5-M-9.5-3		9.5	0.164	1.182	0.021	34.960	7.412
AC-5-M-8.0-4	0.4	8.0	0.218	0.200	0.012	87.245	17.783
AC-5-M-8.5-4		8.5	0.200	0.224	0.016	68.544	16.163
AC-5-M-9.0-4		9.0	0.201	0.270	0.020	66.306	25.760
AC-5-M-9.5-4		9.5	0.237	0.278	0.022	49.755	28.410
AC-5-M-8.0-6	0.6	8.0	0.254	0.115	0.017	89.230	73.940
AC-5-M-8.5-6		8.5	0.249	0.094	0.018	87.627	68.937
AC-5-M-9.0-6		9.0	0.254	0.157	0.020	76.946	56.938
AC-5-M-9.5-6		9.5	0.254	0.146	0.022	71.691	59.583

**Table 4:** Splitting Tensile Strength of Asphalt Mixtures with Varying Gradations and Asphalt Contents, MPa.

**Label**	**Asphalt Content (%)**				
	**7.5**	**8.0**	**8.5**	**9.0**	**9.5**
AC-5-T	-	1.97	1.83	1.77	1.58
AC-5-L		1.49	1.47	1.37	1.83
STRATA-M	2.71	2.16	1.75	1.64	-
**Label**	**Asphalt Content (%)**				
	**3.5**	**3.8**	**4.0**	**4.3**	**4.6**
ATB-25-T	3.45	2.97	2.36	2.78	2.57

**Table 5:** Fatigue Test Results of Asphalt Mixtures with Different Aggregate Gradations and Asphalt Contents.

Label	n	Asphalt Content (%)	ϕ (rad)	$E_{f}$ (MJ/m^3^)	$\varepsilon_{f}$	$S_{f}$ (MPa)	$\dot{\varepsilon }\times 10^{-6}$ (s^−1^)
AC-5-T-8.0-3	0.3	8.0	0.122	0.338	0.016	62.955	11.257
AC-5-T-8.5-3		8.5	0.131	0.731	0.017	48.553	11.152
AC-5-T-9.0-3		9.0	0.134	0.167	0.020	51.702	6.052
AC-5-T-9.5-3		9.5	0.120	0.302	0.024	43.020	66.493
AC-5-L-8.0-3	0.3	8.0	0.145	0.408	0.015	56.897	8.316
AC-5-L-8.5-3		8.5	0.164	0.375	0.015	68.869	9.666
AC-5-L-9.0-3		9.0	0.156	0.609	0.016	49.587	4.923
AC-5-L-9.5-3		9.5	0.148	0.474	0.018	57.815	14.009
STRATA-M-7.5-3	0.3	7.5	0.132	0.283	0.017	91.997	20.421
STRATA-M-8.0-3		8.0	0.151	0.261	0.016	73.773	12.932
STRATA-M-8.5-3		8.5	0.128	0.375	0.020	48.357	9.884
STRATA-M-9.0-3		9.0	0.195	0.250	0.026	35.173	26.384
ATB-25-T-3.5-3	0.3	3.5	0.174	0.543	0.008	261.204	27.283
ATB-25-T-3.8-3		3.8	0.158	0.553	0.010	177.376	17.207
ATB-25-T-4.0-3		4.0	0.156	0.517	0.009	156.136	13.385
ATB-25-T-4.3-3		4.3	0.144	0.837	0.011	145.562	9.497
ATB-25-T-4.6-3		4.6	0.164	0.808	0.011	113.598	12.988

**Table 6:** Sensitivity Analysis of Asphalt Mixture Fatigue Parameters at Varying Stress Levels.

Strain Rate	Parameters	Source of Variance	Sum of Squared Deviations	Degrees of Freedom	Mean Square	F-Value	F_0.05_
0.3	Phase angle	Intergroup	0.002690	3	0.000897	8.966	F_0.05_(3, 8) = 4.07
		Within the group	0.000800	8	0.000100		
		Aggregate	0.003490	11			
	Cumulative dissipated energy	Intergroup	1.095699	3	0.365233	36.523	
		Within the group	0.080000	8	0.010000		
		Aggregate	1.175699	11			
	Failure strain	Intergroup	0.000180	3	0.000060	60.116	
		Within the group	0.000008	8	1.0 × 10^−6^		
		Aggregate	0.000188	11			
	Failure stiffness modulus	Intergroup	1659.19763	3	553.06588	24.581	
		Within the group	180.000000	8	22.500000		
		Aggregate	1839.19763	11			
	Strain rate	Intergroup	36.092625	3	12.030875	27.499	
		Within the group	3.500000	8	0.437500		
		Aggregate	39.592625	11			
0.4	Phase angle	Intergroup	0.000366	3	0.000122	2.995	F_0.05_(3, 8) = 4.07
		Within the group	0.000326	8	0.000041		
		Aggregate	0.000692	11			
	Cumulative dissipated energy	Intergroup	0.007432	3	0.002477	9.16682	
		Within the group	0.002162	8	0.000270		
		Aggregate	0.009594	11			
	Failure strain	Intergroup	0.000119	3	0.000040	20.191	
		Within the group	0.000016	8	0.000002		
		Aggregate	0.000135	11			
	Failure stiffness modulus	Intergroup	2119.208700	3	706.40290	4.161	
		Within the group	1358.000000	8	169.75000		
		Aggregate	3477.208700	11			
	Strain rate	Intergroup	313.610025	3	104.53668	23.230	
		Within the group	36.000000	8	4.500000		
		Aggregate	349.610025	11			
0.6	Phase angle	Intergroup	0.000064	3	0.000021	0.407	F_0.05_(3, 8) = 4.07
		Within the group	0.000418	8	0.000052		
		Aggregate	0.000482	11			
	Cumulative dissipated energy	Intergroup	0.007782	3	0.002594	21.219	
		Within the group	0.000978	8	0.000122		
		Aggregate	0.008760	11			
	Failure strain	Intergroup	0.000037	3	0.000012	5.969	
		Within the group	0.000017	8	0.000002		
		Aggregate	0.000054	11			
	Failure stiffness modulus	Intergroup	642.617700	3	214.20590	6.642	
		Within the group	258.000000	8	32.250000		
		Aggregate	900.617700	11			
	Strain rate	Intergroup	377.091600	3	125.69720	33.519	
		Within the group	30.000000	8	3.750000		
		Aggregate	407.091600	11			

**Table 7:** Sensitivity Analysis of Fatigue Parameters to Asphalt Content Under Varying Stress Ratios.

Strain Rate	Phase Angle	Cumulative Dissipated Energy	Failure Strain	Failure Stiffness Modulus	Strain Rate
0.3	√	√	√	√	√
0.4	×	√	√	√	√
0.6	×	√	√	√	√

**Table 8:** Sensitivity Analysis of Fatigue Parameters Across Aggregate Gradations and Stress Levels.

Graduation Type	Parameters	Source of Variance	Sum of Squared Deviations	Degrees of Freedom	Mean Square	F-Value	F_0.05_
AC-5-T	Phase angle	Intergroup	0.000408	3	0.000136	3.058	F_0.05_(3, 8) = 4.07
		Within the group	0.000356	8	0.000045		
		Aggregate	0.000764	11			
	Cumulative dissipated energy	Intergroup	0.530245	3	0.176745	15.354	
		Within the group	0.092094	8	0.011512		
		Aggregate	0.622339	11			
	Failure strain	Intergroup	0.000118	3	0.000039	54.649	
		Within the group	0.000006	8	7.1828 × 10^−7^		
		Aggregate	0.000124	11			
	Failure stiffness modulus	Intergroup	1190.50403	3	396.8346	89.687	
		Within the group	35.397467	8	4.424683		
		Aggregate	1225.90149	11			
	Strain rate	Intergroup	7364.52080	3	2454.840	889.674	
		Within the group	22.074067	8	2.759258		
		Aggregate	7386.59487	11			
AC-5-L	Phase angle	Intergroup	0.000684	3	0.000228	0.286	F_0.05_(3, 8) = 4.07
		Within the group	0.006372	8	0.000797		
		Aggregate	0.007057	11			
	Cumulative dissipated energy	Intergroup	0.096759	3	0.032253	1.174	
		Within the group	0.219798	8	0.027475		
		Aggregate	0.316558	11			
	Failure strain	Intergroup	0.000022	3	0.000007	1.853	
		Within the group	0.000032	8	0.000004		
		Aggregate	0.000054	11			
	Failure stiffness modulus	Intergroup	569.558967	3	189.8529	3.538	
		Within the group	429.292600	8	53.66157		
		Aggregate	998.851567	11			
	Strain rate	Intergroup	378.466300	3	126.1554	6.954	
		Within the group	145.128467	8	18.14105		
		Aggregate	523.594767	11			
STRATA-M	Phase angle	Intergroup	0.008692	3	0.002897	3.345	F_0.05_(3, 8) = 4.07
		Within the group	0.006929	8	0.000866		
		Aggregate	0.015621	11			
	Cumulative dissipated energy	Intergroup	0.029068	3	0.009689	1.319	
		Within the group	0.058789	8	0.007349		
		Aggregate	0.087857	11			
	Failure strain	Intergroup	0.000184	3	0.000061	34.043	
		Within the group	0.000014	8	0.000002		
		Aggregate	0.000198	11			
	Failure stiffness modulus	Intergroup	5831.39843	3	1943.799	45.873	
		Within the group	338.986067	8	42.37325		
		Aggregate	6170.38450	11			
	Strain rate	Intergroup	498.965700	3	166.3219	7.776	
		Within the group	171.117000	8	21.38962		
		Aggregate	670.082700	11			
ATB-25-T	Phase angle	Intergroup	0.001449	4	0.000362	0.651	F_0.05_(4, 10) = 3.48
		Within the group	0.005568	10	0.000557		
		Aggregate	0.007017	14			
	Cumulative dissipated energy	Intergroup	0.270490	4	0.067623	7.695	
		Within the group	0.087873	10	0.008787		
		Aggregate	0.358364	14			
	Failure strain	Intergroup	0.000028	4	0.000007	3.712	
		Within the group	0.000019	10	0.000002		
		Aggregate	0.000047	14			
	Failure stiffness modulus	Intergroup	37,017.9608	4	9254.490	86.031	
		Within the group	1075.71313	10	107.5713		
		Aggregate	38,093.6740	14			
	Strain rate	Intergroup	560.484640	4	140.1211	24.141	
		Within the group	58.042000	10	5.804200		
		Aggregate	618.526640	14			

**Table 9:** Sensitivity Analysis of Fatigue Parameters to Asphalt Content Across Various Aggregate Gradations.

Graduation Type	Phase Angle	Cumulative Dissipated Energy	Failure Strain	Failure Stiffness Modulus	Strain Rate
AC-5-T	×	√	√	√	√
AC-5-L	×	×	×	×	√
STRATA-M	×	×	√	√	√
ATB-25-T	×	√	√	√	√

**Table 10:** Strain Rate and Fatigue Life of AC-5-M Asphalt Mixtures Under Various Stress Levels and Asphalt Contents.

Stress Level	Asphalt Content (%)	$\dot{\varepsilon }\times 10^{-6}$ (s^−1^)					Fatigue Life (Count)				
		1#	2#	3#	SD	CV (%)	1#	2#	3#	SD	CV (%)
0.3	8.0	3.32	3.82	2.82	0.5	15.1	24,977	19,977	29,977	5000	20.0
	8.5	4.30	3.80	4.80	0.5	11.6	22,480	17,480	27,480	5000	22.2
	9.0	3.04	2.54	3.54	0.5	16.4	40,060	45,060	35,060	5000	12.5
	9.5	7.41	7.41	7.41	0	0	22,019	21,019	23,019	1000	4.5
0.4	8.0	17.78	20.78	14.78	3.0	16.9	3965	4265	3665	300	7.6
	8.5	16.16	17.16	15.16	1.0	6.2	3910	4110	3710	200	5.1
	9.0	27.76	29.76	25.76	2.0	7.2	5270	5350	5190	80	1.5
	9.5	26.41	28.41	24.41	2.0	7.6	4690	4800	4580	110	2.3
0.6	8.0	67.94	70.94	64.94	3.0	4.4	1612	1732	1492	120	7.4
	8.5	68.94	69.94	67.94	1.0	1.5	1894	1904	1884	10	0.5
	9.0	56.94	57.94	55.94	1.0	1.8	2320	2450	2190	130	5.6
	9.5	57.58	59.58	55.58	2.0	3.5	2080	2140	2020	60	2.9

**Table 11:** Strain Rate and Fatigue Life Test Results for Asphalt Mixtures at a Stress Level of 0.3.

Graduation Type	Asphalt Content (%)	$\dot{\varepsilon }\times 10^{-6}$ (s^−1^)					Fatigue Life (Count)				
		1#	2#	3#	SD	CV (%)	1#	2#	3#	SD	CV (%)
AC-5-T	8.0	14.05	8.46	11.26	2.79	24.8	6941	8559	7750	809	10.4
	8.5	11.15	12.15	10.15	1.00	9.0	7235	8235	6235	1000	13.8
	9.0	4.68	7.42	6.05	1.37	22.6	20,679	13,687	17,183	3496	20.3
	9.5	65.90	67.08	66.49	0.59	0.9	2446	1801	2123	322	15.2
AC-5-L	8	6.14	4.57	14.24	5.18	62.4	15,361	18,081	8756	4795	34.1
	8.5	14.33	19.19	25.48	5.59	28.4	18,893	15,061	15,263	2156	13.1
	9.0	4.23	7.57	2.97	2.37	48.3	23,854	14,446	25,437	5941	28.0
	9.5	16.96	11.05	14.01	2.95	21.1	5739	10,971	8355	2616	31.3
STRATA-M	7.5	15.68	18.24	27.35	6.13	30.0	4333	5193	5779	727	14.3
	8.0	13.55	12.31	12.93	0.62	4.8	6942	7123	2400	2676	48.8
	8.5	7.78	8.56	13.31	2.99	30.3	10,390	12,126	7454	2361	23.6
	9.0	24.12	33.41	21.62	6.21	23.5	5553	4261	6069	931	17.6
ATB-25-T	3.5	27.28	28.28	26.28	1.00	3.7	1851	2151	1551	300	16.2
	3.8	17.11	17.30	17.21	0.09	0.6	3472	3093	3282	189	5.8
	4.0	12.92	17.24	10.00	3.64	27.2	3834	4015	4262	214	5.3
	4.3	9.50	10.50	8.50	1.00	10.5	5768	6768	4768	1000	17.3
	4.6	9.09	13.40	16.47	3.70	28.5	6559	3846	4277	1457	29.8

**Table 12:** PCC Calculations for Strain Rate and Fatigue Life of Asphalt Mixtures.

Stress level	0.3		0.4	0.6	
PCC	−0.550		−0.958	−0.718	
Gradation type	AC-5-T	AC-5-M	AC-5-L	STRATA-M	ATB-25-T
PCC	−0.723	−0.550	−0.903	−0.552	−0.868

**Table 13:** Aggregate Gradation Used for the Confirmatory Fatigue Performance Tests of Asphalt Mixtures.

Gradation	Percentage for Passing the Sieve (%)							
	9.50	4.75	2.36	1.18	0.60	0.30	0.15	0.075
	100.0	91.6	64.9	43.5	33.1	21.2	13.9	7.4

**Table 14:** Volumetric Parameters of Asphalt Mixtures Designed Using the Marshall Method.

Index	Asphalt Content (%)				
	7.5	8.0	8.5	9.0	9.5
Bulk density (g/cm^3^)	2.37	2.36	2.36	2.34	2.33
VV (%)	2.51	2.25	1.75	1.61	1.48
VMA (%)	18.92	19.72	20.32	21.20	22.07
VFA (%)	86.77	88.61	91.40	92.41	93.31
MS (kN)	16.41	14.87	12.42	13.15	11.41
FL (0.1 mm)	5.73	7.25	7.44	8.30	9.15

**Table 15:** Volumetric Parameters of Asphalt Mixtures Designed Using the Strain Rate Method.

Index	Asphalt Content (%)				
	7.9	8.2	8.5	8.8	9.1
Bulk density (g/cm^3^)	2.23	2.29	2.29	2.31	2.33
VV (%)	4.23	3.84	3.36	2.10	1.14
VMA (%)	22.73	21.80	22.21	21.48	21.06
VFA (%)	81.97	82.12	82.41	89.90	94.61
$\dot{\varepsilon }\times 10^{-6}$	12.6	10.2	8.5	9.3	14.7

## Data Availability

The data used to support the findings of this study are included within the article.
